# Impact of Implementing Teleophthalmology Referral Guidelines Using the eyeSmart EMR App in 63,703 Patients from India

**DOI:** 10.1155/2022/8523131

**Published:** 2022-01-27

**Authors:** Anthony Vipin Das, Rohit C. Khanna, Niranjan Kumar, Padmaja Kumari Rani

**Affiliations:** ^1^Department of eyeSmart EMR & AEye, L V Prasad Eye Institute, Hyderabad, Telangana, India; ^2^Department of Teleophthalmology, L V Prasad Eye Institute, Hyderabad, Telangana, India; ^3^Gullapalli Pratibha Rao International Centre for Advancement of Rural Eye Care, L V Prasad Eye Institute, Hyderabad, Telangana, India; ^4^Smt Kanuri Santhamma Centre for Vitreo Retinal Diseases, L V Prasad Eye Institute, Hyderabad, Telangana, India

## Abstract

**Objective:**

To describe the clinical indications and the impact of implementation of specific teleophthalmology referral guidelines in a large rural village vision centre network in India.

**Methods:**

This cross-sectional vision centre-based study included 1,016,284 patients presenting between January 2017 and March 2020. Patients who were referred for a teleophthalmology opinion were included as cases. The data were collected using the eyeSmart EMR app on a smart tablet. A training intervention was done to reinforce the implementation of targeted teleophthalmology referral guidelines.

**Results:**

Overall, 63,703 (6.3%) patients were referred for a teleophthalmology opinion and were included for analysis. The median age was 41 (IQR: 26-59) years, and adults (88.4%) were commonly referred for a consult. The two most common age groups were between 31-40 years (17.4%) and 21-30 years (16.3%), and the majority of patients were male (59.1%). The most common clinical indication was cornea and anterior segment disorders (71.05%). The most common queries for teleophthalmology referral before versus after the reinforcement of implementation of guidelines were red eye (33.4% vs. 45.6%) followed by cataract (21.2% vs. 8.1%). There was an increase in the red eye (<0.001) and a decrease in cataract (<0.001) which was statistically significant. The proportion of patients for whom a teleophthalmology consult could have been requested but not sent was minimal (2.3%).

**Conclusion:**

Implementation of targeted teleophthalmology referral guidelines enables an effective triage to seek opinion for more pertinent ocular diseases that require care. Adult male patients with cornea and anterior segment disorders are most commonly referred for a teleophthalmology opinion.

## 1. Introduction

Teleophthalmology is an important tool to provide comprehensive primary eye care bridging the gaps of distance, expertise at remote rural locations. Several studies have reported the accuracy, cost-effectiveness, and patient satisfaction of the teleophthalmology model [1–3]. Barriers to the accessibility of eye care in terms of geography, time, travel, and money have been effectively addressed through effective telemedical systems worldwide [4, 5]. Teleophthalmology models were much more cost-effective than in-person consultations in conditions such as diabetic retinopathy and glaucoma [6, 7]. Telemedicine collaborations between developed and developing countries have shown significant benefits in the provision of expert care with positive results in conditions such as retinoblastoma [8, 9].

Referral guidelines of teleconsultation are crucial for a primary eye care provider to provide the appropriate management and necessary referrals to secondary/tertiary levels of care. Adherence to teleconsultation referral protocols is necessary to provide effective primary eye care. However, the lack of adherence to teleconsultation referral guidelines can result in unnecessary referrals to teleconsultation. It can also result in the missing out of referable pathology by the vision technician. This can be inconvenient for the patient who might incur additional, unnecessary expenses.

Our earlier pilot study of a small sample (5,604 patients) over a limited time period (1 week) showed that around 9% missed referrals for teleconsultation [10].

There was a need to see actual missed referrals in the entire cohort over longer duration and the impact of implementing targeted teleophthalmology referral guidelines.

The present study is designed to understand the adherence patterns to teleconsultation referral guidelines in a primary eye care network through an audit, before and after the implementation of targeted teleophthalmology referral guidelines. The findings of the study will aid in providing holistic primary eye care and the effective implementation of the teleophthalmology model.

## 2. Materials and Methods

### 2.1. Study Design, Period, Location, and Approval

This cross-sectional observational hospital-based study included all patients presenting between January 2017 and March 2020 to the village vision centres of an ophthalmology network located in 176 different geographical locations spread across 4 states (Telangana, Andhra Pradesh, Odisha, and Karnataka) of India [11]. The prevalence of disabled persons in seeing according to the 2011 census of India in erstwhile combined Andhra Pradesh (Andhra Pradesh+Telangana) was 0.47% (398,144/84,580,777), Odisha was 0.63% (263,799/41,974,218), and Karnataka was 0.43% (264,170/61,095,297) [12]. The patient or the parents or guardians of the patient filled out a standard consent form for electronic data privacy at the time of registration. None of the identifiable parameters of the patient information was used for analysis of the data. The study adhered to the Declaration of Helsinki and was approved by the Institutional Ethics Committee (Hyderabad Eye Research Foundation, Hyderabad, Telangana, India). The clinical data of each patient who underwent a comprehensive ophthalmic examination was entered into an app-based electronic medical record system (eyeSmart EMR) [13] by a uniformly trained ophthalmic vision technician using a standardized template that synchronizes to the main EMR system through the cloud [14]. The vision technicians (VT) undergo a year of training at the centre of excellence and are then posted in the respective vision centres across the network. Continuous medical education modules and assessments of the vision technicians are conducted periodically through the app as well. Patients were also referred for a teleophthalmology consult through the eyeSmart app [15] and the anterior segment pictures of the eye taken by a slit lamp attachment [16]. The video conferencing tool, Skype, is used for all the teleophthalmology consults (Skype, Microsoft Corp, Redmond, USA). For optimum patient management and communication with the higher centres, a referral system is put into place whereby the VT decides on either direct referrals or teleconsultations using the eyeSmart app. The teleophthalmology advice is given by an ophthalmologist who is present at the command centre located at the tertiary eye hospital. An ophthalmologist provides advice through electronic medical record after assessing the patient complaints through electronic record data and real-time examination using video call (Skype).

### 2.2. Cases

A total of 1,016,284 patients of all ages presented to the 176 vision centres of the network during the study period. Standard teleophthalmology referral guidelines were formulated which included (a) any lid abnormality such as ptosis, severe meibomitis, blepharitis, stye, chalazion, and other conditions that are obvious on torch light examination, (b) pterygium crossing the limbus, (c) pigmentation of conjunctiva or any abnormality that is clearly seen on a slit lamp, (d) red eye with or without discharge, (e) corneal scars, (f) corneal infiltrates, (g) corneal epithelial defects (need images with and without staining), (h) severe spheroidal degeneration that is close to pupillary margin or covering the pupillary area, (i) shallow anterior chamber (showing the slit view), (j) if the patient has hyperopia more than 3.0 Ds (slit view to be shown), (k) flare and cells, (l) pupil/iris abnormalities such as atrophic patches on the iris, sphincter atrophy, new blood vessels on the iris, (m) ocular trauma, (n) any lens-related pathology which needed teleconsultation, and (o) suspected posterior segment pathology. The patients could also be referred to a higher centre for medical or surgical care.

Primary eye care providers (vision technicians) were instructed to take images as per the standard protocol of teleconsultation of the institute of all patients visiting vision centres. Currently, LVPEI anterior segment image teleconsultation protocol includes an external photo, one anterior segment image of each eye. All vision technicians have been trained in teleconsultation referral guidelines through lectures, PowerPoint slides, and WhatsApp communication periodically. These specific referral guidelines were reinforced through training programs in November 2019, and the reasons for teleophthalmology referral were compared before and after the training intervention.

The patients were classified into the following three categories: (i) patients in whom a teleophthalmology consult was sent (Category 1); (ii) patients who were referred to the secondary centre for higher care through teleophthalmology (Category 2); (iii) patients in whom the teleophthalmology consult could have been sent but was missed. These were identified based on the teleophthalmology referral guidelines (Category 3). All these categories have been compared before and after training intervention.

### 2.3. Data Retrieval and Processing

The data of 1,016,284 patients included in this study were retrieved from the electronic medical record database from the eyeSmart app and segregated in a single excel sheet. The columns included the data on demographics, clinical presentation, ocular diagnosis, and reasons for teleophthalmology referral which were exported for analysis. The excel sheet with the required data was then used for analysis using the appropriate statistical software.

### 2.4. Statistical Analysis

Descriptive statistics using mean ± standard deviation and median with interquartile range (IQR) were used to elucidate the demographic data. The chi-square test (StataCorp. 2015. Stata Statistical Software: Release 14. College Station, TX: StataCorp LP) was used for univariate analysis to detect significant differences in the reasons for referring patients for a teleophthalmology consult.

## 3. Results

### 3.1. Overall Cohort

**Figure 1:**
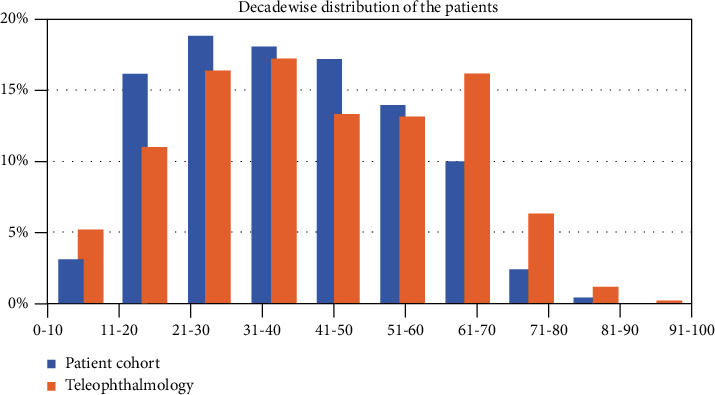
Decade-wise distribution of the overall cohort, teleophthalmology consults.

A total of 1,016,284 patients presented to the village vision centre network during the study period. The mean age of the patients was 38.9 ± 18.0 years while the median age was 38 (IQR: 24-53) years. There were 124,327 (12.2%) children (≤16 years) and 891,957 (87.8%) adults. There were 560,331 (55.1%) male patients and 455,953 (44.9%) female patients. The detailed decade-wise prevalence of patients is summarized in Figure 1. The most common ocular diagnosis were related to cornea and anterior segment in 486,739 (47.9%) patients, refractive error in 346,850 (34.1%) patients, and cataract in 171,244 (16.9%) patients.

### 3.2. Teleophthalmology Cohort (Category 1)

A teleophthalmology consult was sought in 63,703 (6.3%) patients from the village vision centre network during the study period. The mean age of the patients was 41.5 ± 19.6 years while the median age was 41 (IQR: 26-59) years. There were 7,406 (11.6%) children (≤16 years) and 56,297 (88.4%) adults. There were 37,668 (59.1%) male patients and 26,035 (40.9%) female patients. The detailed decade-wise prevalence of patients who were sent for teleophthalmology consultation is summarized in Figure 1. The most common ocular diagnosis were related to cornea and anterior segment in 45,261 (71.1%) patients, cataract in 11,700 (18.4%) patients, and oculoplasty related in 2,238 (3.5%) patients.

Overall cohort versus teleophthalmology cohort: Figure 1 shows comparison of decade-wise prevalence percentages between the overall cohort and teleophthalmology cohort. At 21-40 years (34%) and with increasing age more than 50 years, teleophthalmology referral is significantly more 36.8% (Tele) vs. 26.7% ((overall) *p* = <0.00001).

### 3.3. Patients Who Were Referred to the Secondary Centre for Higher Care through Teleophthalmology (Category 2)

In Table 1, a total of 30,268/63,703 (47.5%) were referred to a higher centre for medical or surgical care from the village vision centre network through teleophthalmology during the study period. The mean age of the patients was 49.2 ± 19.7 years while the median age was 54 (IQR 35-66) years. There were 2,463 (8.1%) children (≤16 years) and 27,805 (91.9%) adults. There were 17,007 (56.2%) male patients and 13,261 (43.8%) female patients. About 5,762 (19%) patients who were referred from the vision centre through teleophthalmology reported to the higher centre for care. The detailed ocular diagnosis referral distribution before and after implementation is shown in Table 2.

### 3.4. Patients in Whom the Teleophthalmology Consult Could Have Been Sent but Was Missed (Category 3)

Overall, there was a small subset of 23,809 (2.3%) patients who could have been referred for a teleophthalmology consult and was not sent by the vision technician. The detailed causes in these patients before and after implementation of specific referral guidelines is described in Table 3. About 2.6% of patients (*n* = 21,429/831,574) were missed referral for teleconsultation before implementation compared to 2% (*n* = 2414/121,007) (*p* value < 0.00001) after implementation.

### 3.5. Impact of Teleophthalmology Referral Guidelines

A total of 50,000 (78.5%) of teleophthalmology consults were done before the implementation of the specific teleophthalmology referral guidelines, and 13,703 (21.5%) were performed after November 2019. The most common queries for teleophthalmology referral before versus after the implementation of guidelines were red eye (33.4% vs. 45.6%) followed by cataract (21.17% vs 8.13%). There was an increase in the red eye (<0.00001) referral and a decrease in cataract (<0.00001) referral for teleophthalmology consults which was statistically significant. The detailed comparison of the ocular diagnosis before and after the implementation of the teleophthalmology guidelines is described in Table 3.

## 4. Discussion

The present study data shows that a technology-enabled primary eye care network could provide effective eye care to a million patients in three years. At the vision centre level, the vision technician utilizes the three “R's” which are Refraction, Recognize (the sight-threatening conditions), and Refer (them to the appropriate higher levels of eye care) [11].

Our network vision technician accuracy in the detection of referable anterior segment pathology has been proven [17]. In the present study, more than 90% of people who visited vision centres could be given necessary eye care at the vision centre itself. About 6.3% required targeted referral for advice through teleophthalmology. Amongst those referred for teleophthalmology advice, more than 50% could be managed solely by teleconsultation advice at the vision centre. These patients could be given treatment advice without the need for travel to a secondary centre. A total of 30,268/63,703 (47.5%) were referred to a higher centre for medical or surgical care from the village vision centre network through teleophthalmology during the study period. Avoiding unnecessary travel shows the advantage of teleophthalmology in providing economic impact as well as the environmental impact on reducing carbon footprint. Telemedicine has the potential to build a carbon-free health system by reducing carbon emissions associated with travel. In a systematic survey by Purohit et al., carbon footprint savings ranged from 0.70 to 372 kg of CO_2_ per consultation [18].

Our study results (Figure 1) shows that with patients of more than 50 years of age, the teleophthalmology referral was significantly more compared to their overall cohort (36.7% vs. 26% *p* ≤ 0.00001). This finding is important as older individuals could get initial advice at the primary care location through teleophthalmology without the need to travel to farther locations. This is the advantage of the teleophthalmology model which can address the barrier of distance and travel effectively.

Cornea and anterior segment pathology and oculoplasty-related pathology were the predominant conditions that were given teleophthalmology care. Teleophthalmology efficacy in the detection and management of ocular surface and adnexal diseases is well proven [10, 19, 20]. Komal et al. clearly showed the cost benefits in treating corneal conditions alone through teleophthalmology were about 1200 INR (16 US dollars) per patient; total cost saving for the community for one-year duration was 1.14 million rupees and 15,000 US dollars [5].

A slightly more number of male patients sought eye care overall and in the teleophthalmology cohort. This gender inequality in the access to eye care needs to be addressed by a root cause analysis. Similarly, it was observed that around 20% only reached higher care centres after referrals from the teleophthalmology model. The reasons for this suboptimal reach need to be explored in future studies.

There is paucity of literature regarding teleophthalmology referral guidelines and adherence patterns. The major finding of the present study is that there was more than 98% adherence to the teleophthalmology referral guidelines by the vision technicians. Only 2.3% of one million cohort patients missed their referral for a potential teleophthalmology advice. The comparison of these missed numbers, before and after training, showed that there was a significant decrease in the missed referrals for teleconsultation after training and reinforcing targeted teleophthalmology referral guidelines (2.6% vs. 2%, *p* < 0.00001).

Another important objective of the present study was to find the impact of reinforcing the targeted teleophthalmology referral guidelines through training of vision technicians. The most common queries for teleophthalmology referral before the reinforcement of guidelines were red eye (33.4% vs. 45.6%) followed by cataract (21.2% vs. 8.1%). There was an increase in the red eye (<0.00001) and a decrease in cataract (*r* < 0.00001) which was statistically significant. This is important as a routine cataract does not need teleophthalmology advice and can be directly referred to a secondary centre for appropriate care, whereas a diagnosis of red eye can be effectively managed by teleconsultation alone. The major strengths of the study are the use of digital systems at the rural eye care level enables us to track the efficiency of the delivery of eye care services. The continuous monitoring of the adherence to the teleophthalmology guidelines by retrieving the digital data captured through the eyeSmart EMR app enables positive reinforcement and decreases the burden on the need for unnecessary referrals. The study in this large cohort of patients also lends insight that all patients who present to the vision centre do not require a teleophthalmology consult thereby catering to the patients who actually need the second opinion. The limitations of the study are user variability in the referral pattern across the vision centres. This is accountable to a fair extent by the training provided to them for a year before deployment and also the reinforcement of the teleophthalmology referral guidelines. The users also have a learning curve in the use of the eyeSmart EMR app and the protocol to send the pictures to the command centre. The preexisting knowledge about the use of smart phone apps by them helped to a great extent in the quick adaptation to this new technology.

To conclude, inbuilt audits and periodic training interventions to improve the adherence to targeted teleophthalmology guidelines are essential components in the implementation of the successful teleophthalmology model for effective primary eye care.

## Figures and Tables

**Table 1:** Patients who were referred to the secondary centre for higher care through teleophthalmology (Category 2).

Specialty	Before		After	
	25723/50,000	84.98%	4545/13,703	15.02%
Cornea & anterior segment	13219	51.39%	3053	67.17%
Cataract	9911	38.53%	923	20.31%
Trauma	924	3.59%	171	3.76%
Refractive error	766	2.98%	159	3.50%
Oculoplasty	399	1.55%	153	3.37%
Retina	286	1.11%	39	0.86%
Glaucoma	102	0.40%	27	0.59%
Neuroophthalmology	46	0.18%	10	0.22%
Uvea	38	0.15%	8	0.18%
Strabismus	32	0.12%	2	0.04%

**Table 2:** Ocular diagnosis distribution in patients whose teleophthalmology consultation was missed and not sent (Category 2).

Diagnosis	Before		After	
	21429/23803	89.88%	2414/23803	10.12%
Ocular surface	11830	49.62%	1406	5.90%
Pterygium	8733	36.63%	1058	4.44%
Allergic eye disease	1519	6.37%	203	0.85%
Pinguecula	868	3.64%	87	0.36%
Subconjunctival haemorrhage	551	2.31%	53	0.22%
Conjunctival abrasion	118	0.49%	3	0.01%
Episcleritis	16	0.07%	2	0.01%
Conjunctival pigmentation	13	0.05%	na	na
Bitot spots	12	0.05%	na	na
Eyelid	3042	12.76%	310	1.30%
Meibomitis	1750	7.34%	173	0.73%
Stye	927	3.89%	96	0.40%
Chalazion	250	1.05%	30	0.13%
Ptosis	60	0.25%	9	0.04%
Blepharitis	55	0.23%	2	0.01%
Cornea	2413	10.12%	224	0.94%
Corneal scar	938	3.93%	98	0.41%
Corneal foreign body	873	3.66%	43	0.18%
Corneal abrasion	190	0.80%	29	0.12%
Keratitis	123	0.52%	25	0.10%
Epithelial defect	119	0.50%	15	0.06%
Spheroidal degeneration	112	0.47%	12	0.05%
Corneal edema	43	0.18%	2	0.01%
Superficial punctate keratitis	15	0.06%	na	na
Anterior segment	1532	6.43%	165	0.69%
Red eye	1532	6.43%	165	0.69%
Neuroophthalmology	1188	4.98%	144	0.60%
Pupillary abnormality	1141	4.79%	135	0.57%
Optic nerve pathology	47	0.20%	9	0.04%
Retina	442	1.85%	33	0.14%
Suspect retinal problem	386	1.62%	21	0.09%
Coloboma	55	0.23%	11	0.05%
Retinitis pigmentosa	1	0.00%	1	0.00%
Strabismus	434	1.82%	70	0.29%
Strabismus	434	1.82%	70	0.29%
Glaucoma	351	1.47%	35	0.15%
Glaucoma suspect	281	1.18%	23	0.10%
Shallow AC	68	0.29%	12	0.05%
Acute angle closure	2	0.01%	na	na
Trauma	166	0.70%	21	0.09%
Trauma	166	0.70%	21	0.09%
Uvea	31	0.13%	31	0.13%
Uveitis	31	0.13%	31	0.13%

**Table 3:** Comparative description of ocular diagnosis referral before and after the implementation of specific teleophthalmology referral guidelines.

Ocular diagnosis	Before		After		*p* value
	*N*	%	*N*	%	
Allergic eye disease	2076	4.15%	800	5.84%	*<0.00001*
Bitot spots	53	0.11%	17	0.12%	0.572284
Blepharitis	231	0.46%	63	0.46%	0.972703
Cataract	10586	21.17%	1114	8.13%	*<0.00001*
Chalazion	386	0.77%	119	0.87%	0.263399
Coloboma	30	0.06%	6	0.04%	0.479456
Conjunctival abrasion	341	0.68%	23	0.17%	*<0.00001*
Corneal abrasion	312	0.62%	62	0.45%	*0.020548*
Corneal epithelial defect	2221	4.44%	758	5.53%	*<0.00001*
Corneal foreign body	1093	2.19%	289	2.11%	0.59175
Corneal scar	1302	2.60%	330	2.41%	0.210135
Corneal ulcer	503	1.01%	136	0.99%	0.889236
Descemet membrane folds	16	0.03%	5	0.04%	0.797699
Episcleritis	22	0.04%	10	0.07%	0.180128
Glaucoma suspect	49	0.10%	12	0.09%	0.726844
Headache	513	1.03%	195	1.42%	*0.000104*
Meibomitis	307	0.61%	78	0.57%	0.551393
Ocular pain	470	0.94%	180	1.31%	*0.000138*
Posterior subcapsular opacification	262	0.52%	32	0.23%	*<0.00001*
Pinguecula	507	1.01%	140	1.02%	0.937374
Pterygium	3569	7.14%	732	5.34%	*<0.00001*
Ptosis	43	0.09%	10	0.07%	0.639735
Pupillary abnormality	60	0.12%	13	0.09%	0.441594
Query	3410	6.82%	968	7.06%	0.350478
Raised IOP	32	0.06%	10	0.07%	0.717026
Red eye	16693	33.39%	6252	45.63%	*<0.00001*
Shallow anterior chamber	30	0.06%	9	0.07%	0.811915
Spheroidal degeneration	121	0.24%	21	0.15%	0.051442
Superficial punctate keratitis	236	0.47%	84	0.61%	*0.039661*
Strabismus	37	0.07%	4	0.03%	0.06704
Stye	1274	2.55%	406	2.96%	*0.009*
Subconjunctival haemorrhage	1403	2.81%	414	3.02%	0.192758
Suspect retinal problem	297	0.59%	47	0.34%	*0.000407*
Trauma	1470	2.94%	350	2.55%	*0.01946*
Uveitis	45	0.09%	14	0.10%	0.678561
Grand total	50000	100.00%	13703	100.00%	

## Data Availability

Access to data is restricted.
